# A Review of Weak Gel Fracturing Fluids for Deep Shale Gas Reservoirs

**DOI:** 10.3390/gels10050345

**Published:** 2024-05-18

**Authors:** Shichu Yang, Weichu Yu, Mingwei Zhao, Fei Ding, Ying Zhang

**Affiliations:** 1 College of Chemistry & Environmental Engineering, Yangtze University, Jingzhou 434023, China; 2021710206@yangtzeu.edu.cn (S.Y.); yuweichu@126.com (W.Y.); 2 Hubei Engineering Research Centers for Clean Production and Pollution Control of Oil and Gas Fields, Jingzhou 434023, China; 3 State Key Laboratory of Deep Oil and Gas, China University of Petroleum (East China), Qingdao 266580, China; zhaomingwei@upc.edu.cn (M.Z.); df_1206@163.com (F.D.)

**Keywords:** deep shale gas, viscosity-controllable drag reducer, synthesis method, application status

## Abstract

Low-viscosity slickwater fracturing fluids are a crucial technology for the commercial development of shallow shale gas. However, in deep shale gas formations with high pressure, a higher sand concentration is required to support fractures. Linear gel fracturing fluids and crosslinked gel fracturing fluids have a strong sand-carrying capacity, but the drag reduction effect is poor, and it needs to be pre-prepared to decrease the fracturing cost. Slick water fracturing fluids have a strong drag reduction effect and low cost, but their sand-carrying capacity is poor and the fracturing fluid sand ratio is low. The research and development of viscous slick water fracturing fluids solves this problem. It can be switched on-line between a low-viscosity slick water fracturing fluid and high-viscosity weak gel fracturing fluid, which significantly reduces the cost of single-well fracturing. A polyacrylamide drag reducer is the core additive of slick water fracturing fluids. By adjusting its concentration, the control of the on-line viscosity of fracturing fluid can be realized, that is, ‘low viscosity for drag reduction, high viscosity for sand-carrying’. Therefore, this article introduces the research and application status of a linear gel fracturing fluid, crosslinked gel fracturing fluid, and slick water fracturing fluid for deep shale gas reservoirs, and focuses on the research status of a viscous slick water fracturing fluid and viscosity-controllable polyacrylamide drag reducer, with the aim of providing valuable insights for the research on water-based fracturing fluids in the stimulation of deep shale gas reservoirs.

## 1. Introduction

Global shale gas resources have abundant reserves, with the recoverable resources estimated at 220.69 × 10^12^ m^3^ [1]. Shale gas reservoirs are characterized by a low porosity and permeability, and more than 90% of the resources require fracturing to achieve increased production [2,3,4]. At present, there are many kinds of fracturing fluids used in the development of shale gas, as shown in Table 1. T. S. Yushchenko et al. [5] carried out long horizontal well fracturing in the Bazhenov formation, which significantly improved the production of oil and gas wells. The application of multi-stage fracturing and slickwater fracturing in long horizontal wells has driven the commercial development of shale gas in the middle and shallow layers (buried depth < 3500 m) [6,7,8,9,10]. Slickwater fracturing fluids, known for their low viscosity (<0.005 Pa·s), are widely used in middle and shallow layers due to its ability to create a wide network of fractures in the formation [11].

However, the exploration and development of deep shale gas (buried depth > 3500 m) face challenges, as these reservoirs are characterized by high temperatures and high pressures, leading to fracture closure and poor reservoir stimulation effects. To address this issue, it is necessary to enhance the viscosity and displacement of the fracturing fluid, as well as enhance the fluids’ sand-carrying capacity to effectively support the fractures. Low-viscosity slickwater fracturing fluids alone cannot meet these requirements. As a solution, researchers have proposed a hybrid fracturing fluid consisting of a low-viscosity slickwater fracturing fluid and high-viscosity linear gel/crosslinked gel fracturing fluid: the low-viscosity fluid fractures and the high-viscosity carries the sand. However, there are challenges in the fracturing construction process, such as high construction pressures and difficulties in adding sand, resulting in unsatisfactory production enhancement effects. Additionally, the use of linear gel and crosslinked gel fracturing fluids requires pre-mixing, prolonging the construction cycle and increasing fracturing costs [12].

**Table 1:** Types of fracturing fluids for shale gas development.

Type	Essential Components	Applicable Reservoir	Advantage	Drawback
Oil-based fracturing fluids	Oil, additives	Low permeability and strong water sensitivity reservoirs	High temperature resistance, good lubricity	Harmful to the environment, flammable, high cost
Water-based fracturing fluids	Water, thickeners, additives	Low porosity and low permeability reservoirs	Good sand-carrying performance, low damage, easy flowback	Synthesis cost is high
Foam fracturing fluids	Gas phase (CO_2_, N_2_), liquid phase, additives	Ultra-low permeability reservoirs	High viscosity, low damage, low permeability damage	Poor stability
Acidizing and fracturing fluids [13,14]	Acid (hydrochloric acid, etc.), additives	Low permeability reservoir	Increased permeability and reduced costs	High reservoir damage
CO_2_ fracturing fluids	CO_2_, additives	Low permeability oil layer, water sensitive formations	Reduced wasting of water resources, reduced reservoir damage	Poor sand- carrying performance, high equipment requirements

To overcome these challenges, researchers have developed a “viscosity slickwater fracturing fluid” that combines the advantages of both low-viscosity slickwater fracturing fluids and high-viscosity linear gel fracturing fluids. This hybrid fluid simultaneously extends the main fractures with the linear gel and enters micro-fractures and secondary fractures with the low-viscosity slickwater fluid, facilitating the formation of a complex fracture network and increasing the volume of reservoir transformation. Furthermore, the “viscosity slickwater fracturing fluid” offers the advantages of low viscosity for drag reduction and high viscosity for carrying sand. It does not require pre-mixing, thereby significantly reducing fracturing costs [15].

Polymer drag reducers play a crucial role in slickwater fracturing fluids by reducing drag during high-speed fluid flow. The development of slick fracturing fluids relies on the innovation of drag reducers. Viscosity-controllable slickwater fracturing fluids rapidly adjust the fluid viscosity by changing the concentration of the drag reducer [16]. It can be seen that the study of viscous drag reducers has important practical significance for the efficient development of deep shale gas. Therefore, this article introduces the research and application status of linear gel fracturing fluids, crosslinked gel fracturing fluids, slick water fracturing fluids, and viscous slick water fracturing fluids, and focuses on the synthesis of viscosity-controllable drag reducers from the two aspects of molecular structure and synthesis method, in order to provide theoretical guidance for the research and application of water-based fracturing fluids for deep shale gas reservoir reconstruction.

## 2. Linear Gel Fracturing Fluids

Linear gel fracturing fluids are important working fluids for the hydraulic fracturing of shale gas. They have the advantages of low damage, low friction, and easy flowback. In shale gas accumulation fracturing operations, the linear gel fracturing fluid can play a role in the pre-flush stage for the predicted natural fracture development section.

Linear gel fracturing fluids mainly use a water-soluble linear polymer thickener as the main agent, which can increase the viscosity of the fracturing fluid and improve the sand-carrying performance [17]. Thickeners are mainly divided into natural plant gums and synthetic polymers. The natural plant gums mainly include guanidine gum, xanthan gum, coumarin gum, etc. Natural plant gums and their derivatives have the advantages of a strong thickening ability and easy crosslinking to form a jelly. They have become the most used thickeners in fracturing operations around the world, accounting for about 90% of all the currently used fracturing fluid thickeners. In the 1860s and 1880s, guanidine gum and its derivatives gradually occupied the mainstream [18].

**Figure 1:**
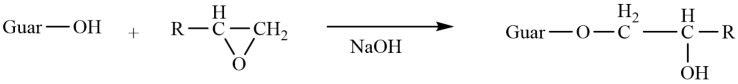
Synthesis route for hydroxypropyl guar gum (HPG) [24].

**Figure 2:**
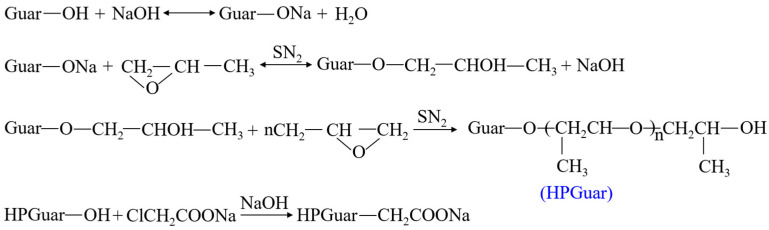
JK-1002 carboxymethyl guanidine gum (CMHPG) preparation schematic diagram [25].

**Figure 3:**
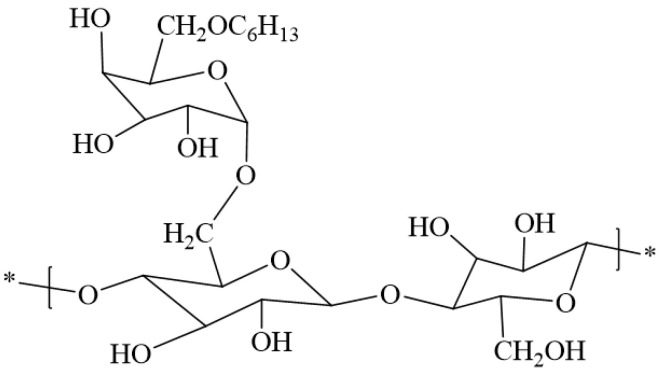
Structure of cationic guar gum [26].

**Figure 4:**
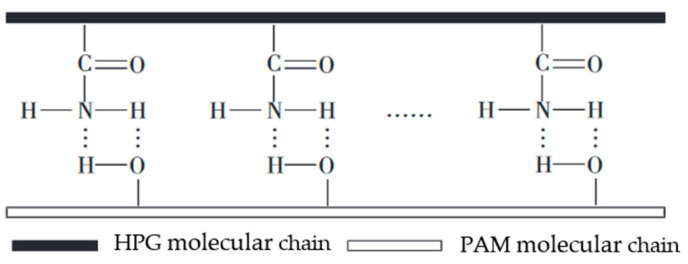
Synthesis principle for high-temperature-resistant composite thickeners [27].

The molecular chain of guanidine collagen powder is mainly composed of galactomannan, which has abundant sources, a low cost, excellent thickening ability, and good temperature and salt tolerance. It is a natural thickener [19]. However, guanidine collagen powder has a slow swelling rate in water, high residue content, and is easily biodegraded [20].The current solutions mainly include hydroxypropylation, carboxymethylation, cationization, and copolymerization with acrylamide [21], as shown in Figure 1, Figure 2, Figure 3 and Figure 4. Among them, hydroxypropyl guanidine gum (HPG) is the most widely used [22,23]. Xiong et al. [24] synthesized an HPG by the phase catalytic method. Compared with guanidine gum, the viscosity of the HPG was significantly better than that of the guanidine collagen powder at 1.0 wt%, which could reach 4.3 Pa·s, and the residue content was lower.

However, the temperature of deep shale gas reservoirs is high, and the glycosidic bonds in the guanidine gum polymer chain are prone to degradation after exceeding 177 °C, resulting in a decrease in the viscosity of the linear gel fracturing fluid. Therefore, the viscosity of the fracturing fluid can only be increased by increasing the amount of fracturing fluid, resulting in an increase in the cost of the fracturing fluid, an increase in the amount of residue, an increase in reservoir damage, an increase in flow friction, and difficulties in fracturing construction [28,29].

## 3. Crosslinked Gel Fracturing Fluids

Crosslinked gel fracturing fluids can be formed by crosslinking thickeners with crosslinking agents such as boron, zirconium, and titanium, which can improve their temperature resistance. The fracturing fluid still has good sand-carrying performance under high temperature conditions [30,31,32]. Crosslinked gel fracturing fluids have excellent temperature and shear resistance and an effective sand-carrying capacity [33]; they are mainly composed of a gel formed by the combination of a thickener and a crosslinking agent [34].

The thickener is the core of crosslinked gel fracturing fluids, with guanidine gum and acrylamide polymers being the most widely studied. In recent years, with the increase in the amount of fracturing fluids needed, the source of guanidine gum is limited and the price of guanidine gum is rising. Acrylamide polymers have received extensive attention due to their low cost and production of less residue. Dai et al. developed a polymer, AAAD, which can be combined with an organic zirconium crosslinking agent to form a gel fracturing fluid. After shearing at 200 °C and 170 s^−1^ for 1 h, the viscosity can be maintained at 0.095 Pa·s [35]. Ma et al. synthesized a heat-resistant polymer as a thickener by solution polymerization, and crosslinked it with an organic zirconium crosslinking agent to prepare an ultra-high-temperature-resistant polymer gel fracturing fluid. After continuous shearing at 200 °C for 1 h, the viscosity could still be maintained at about 0.147 Pa·s, and compared with the guanidine gum fracturing fluid, the core damage rate was significantly reduced [36].

Crosslinking agents are an indispensable additive in crosslinked gel fracturing fluids. They can interact with the crosslinked structural unit in the thickener molecule. By forming different types of chemical bonds, including covalent bonds, ligand bonds, ionic bonds, etc., the linear molecular chain is transformed into a complex spatial network structure, thereby effectively improving the viscosity and temperature resistance of the gel system [37]. At present, crosslinking agents are mainly divided into inorganic crosslinking agents and organic crosslinking agents. Among them, inorganic crosslinking agents often face problems such as a short crosslinking time and uneven crosslinking, and are generally used in middle and shallow shale gas fracturing.

**Figure 5:**
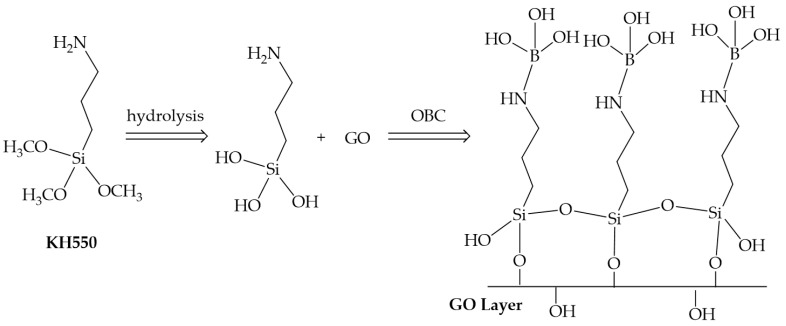
Schematic diagram of GOB synthesis procedure [40].

**Figure 6:**
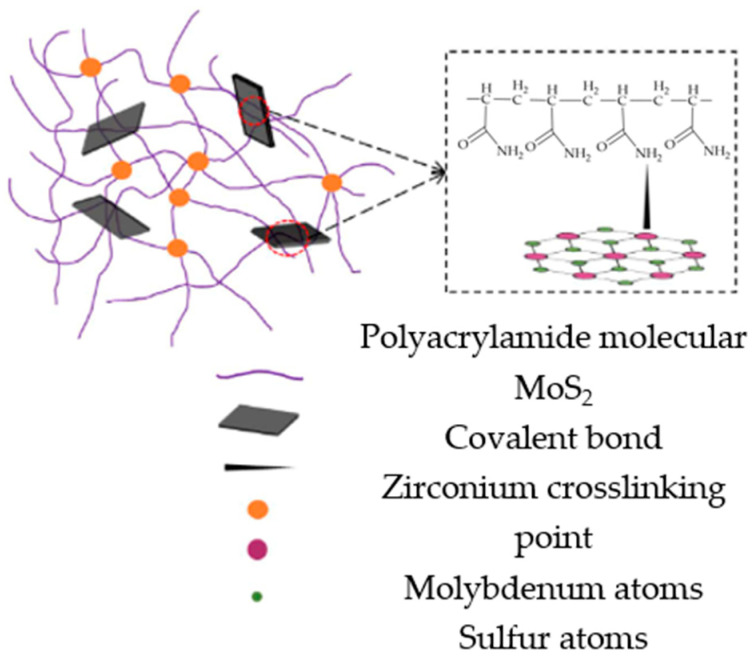
Schematic mechanism of nanocomposite crosslinked fracturing fluid [41].

Due to the needs of deep/ultra-deep fracturing, researchers have proposed organometallic crosslinking agents. Compared with inorganic crosslinking agents, the intermolecular bond energy between organometallic crosslinking agents and thickeners is large, which can greatly improve the temperature resistance of fracturing fluids [38]. Due to the large specific surface area and abundant active hydroxyl groups of nanomaterials, some scholars have prepared nano-crosslinking agents, which can effectively increase the size of crosslinking agents and crosslinking sites, reduce the dosage of thickeners needed, and reduce fracturing costs [39]. Zhang et al. [40] prepared a boron-functionalized colloidal graphene oxide crosslinking agent (GOB), as shown in Figure 5, which effectively improved the temperature resistance and shear resistance of a guanidine gum fracturing fluid. The viscosity of the 0.3 wt% crosslinked gel system was still more than 0.05 Pa·s after shearing at 120 °C and 170 s^−1^. Compared with the conventional organic boron crosslinking agent, the temperature resistance of the fracturing fluid increased by 8.9 °C, and the viscosity increased by 0.02~0.05 Pa·s. Xue et al. [41] combined nano-molybdenum disulfide with the organic zirconium crosslinking agent OZ-60 and polymer thickener CX-200 to prepare a nano-composite crosslinked gel fracturing fluid, as shown in Figure 6. After shearing at 180 °C and 170 s^−1^ for 2 h, the viscosity of the system was still maintained above 0.075 Pa·s.

However, linear gel fracturing fluids and crosslinked gel fracturing fluids need to be prepared in advance, which increases the cost of the fracturing fluid. Under high shear conditions, the gel structure is easily destroyed, which affects the sand-carrying effect of reservoir, and the drag reduction effect is worse than that of slick water fracturing fluids. Therefore, for the fracturing construction of deep shale gas, the pre-flushing fluid generally uses a slick water fracturing fluid system to achieve effective drag reduction, and the subsequent sand-carrying fluid is often a gel with a certain viscosity or a crosslinked linear gel fracturing fluid to achieve effective carrying of sand, which also leads to a complex injection process and high equipment requirements in the fracturing construction process [16].

## 4. Slickwater Fracturing Fluids

### 4.1. Low-Viscosity Slick Water Fracturing Fluids

In 1997, Mitchell Energy used a slickwater fracturing fluid for the first time in Barnett shale in the United States. The recovery rate was increased by 20.0% and the cost was reduced by about 65.0% [42]. Mayerhofer et al. compared and analyzed the fracturing stimulation effect of a slickwater fracturing fluid and linear gel fracturing fluid in the Cotton Valley reservoir in Texas, and found that the slickwater fracturing fluid had a better stimulation effect. The fracturing concepts of ‘self-support’ and ‘single-layer support’ and the fracturing fluid technology concept of a low sand ratio and low viscosity were put forward [42,43]. Subsequently, with the commercial development process of shale gas in the United States, the application of slickwater fracturing fluids gradually increased, and its share in the North American fracturing fluid market exceeded 95% [44].

**Figure 7:**
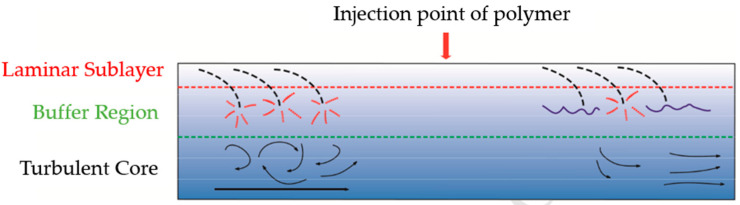
Schematic diagram of drag reduction mechanism of polymer drag reducer [12].

During the injection process of hydraulic fracturing, the fracturing fluid undergoes turbulent flow, resulting in a significant loss of kinetic energy that should have been delivered to the reservoir and leading to insufficient fracturing efficiency [45]. Slickwater fracturing fluids are also known as drag-reducing water fracturing fluids. A polymer drag-reducing agent is the core additive, which can greatly reduce the kinetic energy loss during fracturing. Polymer drag reducers can interact with turbulent eddies by stretching their large polymer molecules, thereby inhibiting the generation, development, and disturbance of turbulent eddies. Moreover, the rebound of coiled polymer molecules can release the dissipative energy accumulated during the eddy and turbulence processes, altering the flow structure and reducing the energy loss. As a result, the drag during the hydraulic fracturing process is significantly reduced, as shown in Figure 7 [12,46].

Based on their source, polymer drag reducers can be classified into natural plant gums and synthetic polymers. The main natural plant gums include guar gum, xanthan gum, and locust bean gum. From the 1960s to the 1980s, guar gum and its derivatives became the mainstream [18]. Among them, hydroxypropyl guar gum (HPG) is the most commonly used [22,23]. However, natural plant gums generally exhibit poor drag reduction effects (<65.0%) and have gradually been replaced by synthetic polymers.

**Figure 8:**
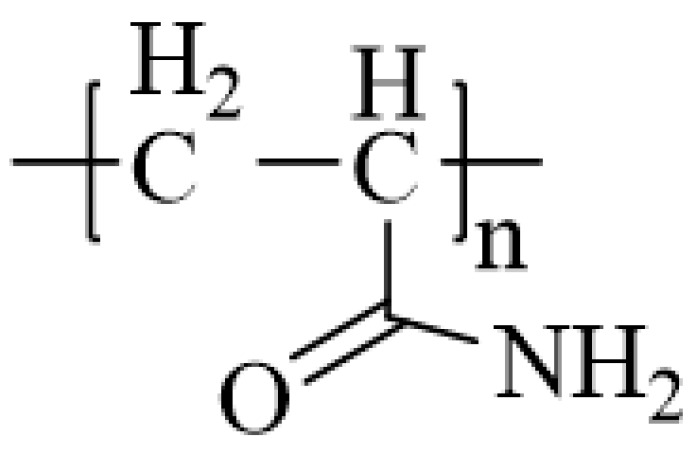
Molecular structure diagram of polyacrylamide.

The synthetic polymer drag reducers that have been extensively investigated are polyethylene glycol, polyethylene oxide, and polyacrylamide [47]. Oliver et al. discovered that a concentration of 3 ppm of polyacrylamide (Figure 8) already demonstrates drag reduction effects [48]. Hoyt compared the drag reduction effects of polyethylene glycol, polyacrylamide, and guar gum in water and found that polyacrylamide exhibited the most favorable performance [49]. Polyacrylamide and its derivatives are cost-effective and exhibit excellent drag reduction performances, thus making them widely utilized as drag reducers.

### 4.2. Viscous Slickwater Fracturing Fluids Similar to Weak Gels

**Figure 9:**
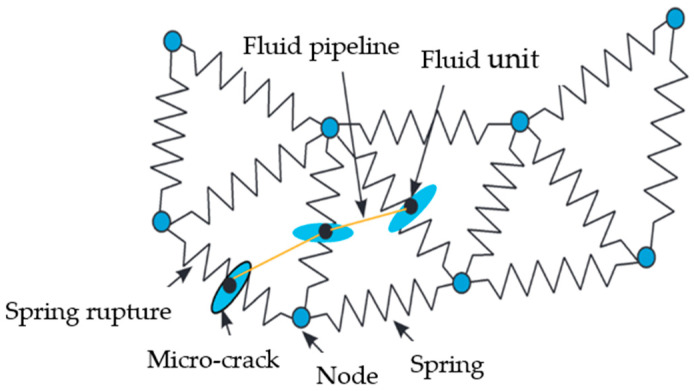
Three-dimensional discrete lattice model [50].

Deep shale gas reservoirs exhibit high closure stress, necessitating the injection of high concentrations of proppants to ensure the effective support of the fracture network. Zhang et al. employed a three-dimensional discrete grid method to construct a numerical model that incorporates bedding effects within an experimental scale of 300 mm × 300 mm × 300 mm, as shown in Figure 9. Their findings indicated that augmenting the viscosity of the fracturing fluid enhances its ability to penetrate and transform fractures in deep shale reservoirs [50].

**Figure 10:**
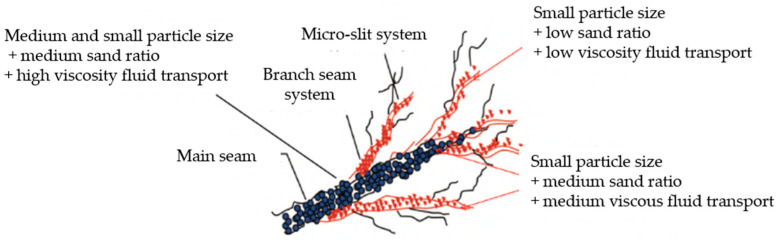
Displacement, proppant particle size, and fracturing fluid viscosity are combined to form different construction stages [53].

Nevertheless, low-viscosity slickwater fracturing fluids exhibit an inadequate proppant-carrying capacity and fail to meet the demands for high-concentration proppant injection. To tackle this issue, Bell et al. introduced the concept of “ULPXLS”, a viscosity-controlled fracturing fluid that fundamentally constitutes a borate/polymer crosslinked system. The fundamental principle underlying ULPXLS is to rapidly increase the viscosity at the surface to enhance the proppant-carrying capacity, while promptly decreasing the viscosity within the fracture to promote the generation of complex fractures [51,52]. However, linear gel fracturing fluids and crosslinked fracturing fluids are associated with drawbacks, such as high cost, suboptimal drag reduction, and reservoir damage. To address these concerns, researchers have developed polyacrylamide-based drag reducers that can dynamically adjust the fluid’s viscosity, exhibiting properties akin to linear gels or weak gels. These drag reducers demonstrate a notable proppant-carrying capacity comparable to that of linear gels or gelled fracturing fluids, along with the proficient drag reduction performance of polyacrylamide. Consequently, they effectively resolve the inherent contradiction between the high drag reduction and low proppant-carrying capacity observed in slickwater fracturing fluids [53]. By manipulating the concentration of the drag reducer, viscosity adjustments of the fracturing fluid can be achieved, fully capitalizing on the strengths offered by fracturing fluids with different viscosities, thereby optimizing the fracture length and fracture network volume while reducing the application costs associated with fracturing fluids, as shown in Figure 10.

Overall, the development of viscosity-controllable polyacrylamide-based DRAs represent a notable advancement in the field of fracturing fluid technology. It offers a more efficient and cost-effective solution to address the challenges associated with enhancing the sand-carrying capacity and drag reduction, ultimately enhancing the performance of slickwater fracturing fluids in operations using high proppant concentrations.

**Figure 11:**
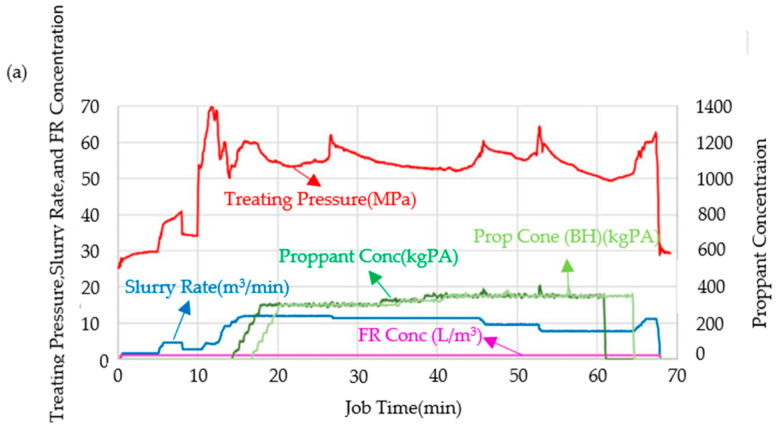

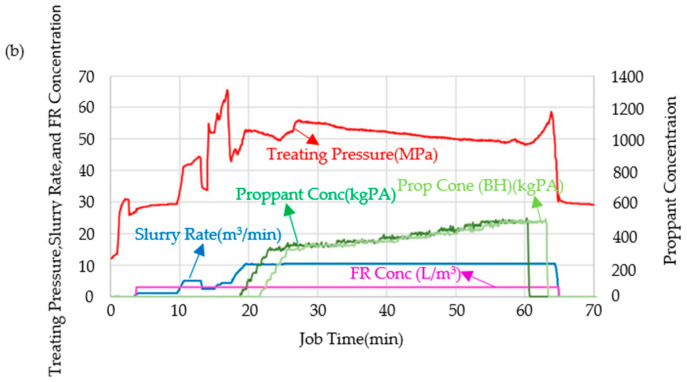
Field construction curves of (**a**) conventional slickwater and (**b**) viscous slickwater [54].

A viscosity-controllable drag reducer can effectively control the viscosity of a fracturing fluid, enabling the development of a viscous slickwater. Zhao et al. [54] successfully synthesized a low-damage and instantly soluble viscosity-controllable polymer drag reducer. At a concentration of 0.5 gpt, the drag reducer achieved a drag reduction rate of 70%. Furthermore, at concentrations ranging from 2 to 4 gpt, the resulting viscosity was comparable to that of a linear gel, ranging from 0.015 to 0.020 Pa·s. Based on these findings, a viscous slickwater technology was developed. Field applications have demonstrated that this technology surpasses conventional slickwater in terms of drag reduction and proppant-carrying capacity, as shown in Figure 11.

**Figure 12:**
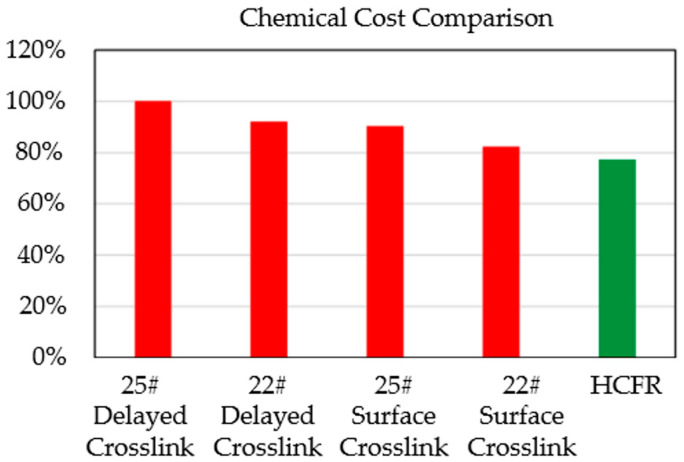
Chemical cost comparison for standard Bakken systems [16].

Motiee et al. [16] used a high concentration polymer drag reducer (HCFR) to simplify the pumping process and formulation of fracturing fluids. This approach resulted in a cost reduction in various aspects, including for chemicals, fracturing equipment, and heating of the fracturing water during the fracturing process. On average, the cost per well was reduced by approximately 22.0%, as shown in Figure 12.

It is evident that viscous slickwater fracturing fluids possess several advantageous characteristics compared to conventional fracturing fluids. These include drag reduction and low damage effects, as well as good proppant placement and flowback. Furthermore, these fluids are easy to formulate and adjust [55]. Additionally, they contribute to cost reductions in fracturing operations and can enhance well productivity.

## 5. Synthesis of Viscosity-Controllable Polyacrylamide Drag Reducers

### 5.1. Synthetic Pathway

To enhance the thickening effect of polyacrylamide in an aqueous solution, the incorporation of hydrophobic groups, highly polar groups, and amphoteric ion groups into the side chains of polyacrylamide molecules can be explored [56].

#### 5.1.1. Grafting Hydrophobic Side Chains

**Figure 13:**
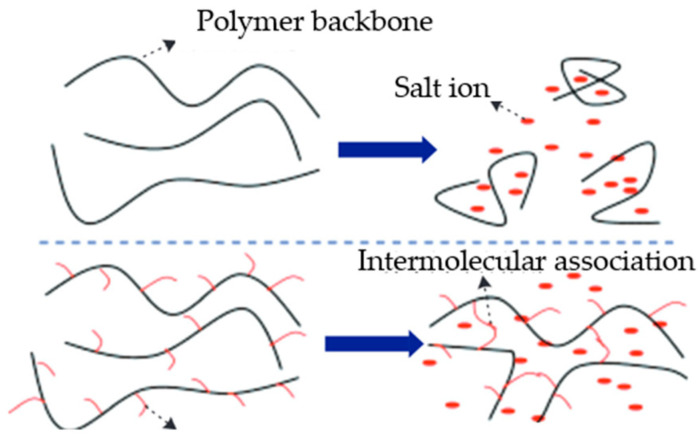
Schematic diagram of the associations between hydrophobic functional monomers [57].

The incorporation of hydrophobic monomers during the copolymerization of acrylamide (AM) allows for the introduction of hydrophobic side chains into the polyacrylamide molecules, enabling the formation of micelles in aqueous solutions through intermolecular and intramolecular associations. Intramolecular associations contribute to increased molecular rigidity, while intermolecular associations lead to the formation of a continuous network structure. Beyond the critical micelle concentration, a supramolecular dynamic network structure is formed, resulting in an increase in hydrodynamic volume and enhanced adsorption bridging of the main chains, thereby significantly enhancing the viscosity of the copolymer solution, as shown in Figure 13 [57].

The commonly employed hydrophobic monomers include long-chain alkyl acrylamide, acrylates, styrene, and long-chain quaternary ammonium salts. Zhou et al. [58] synthesized a ternary copolymer of AM, N-vinylpyrrolidone (NVP), and N-alkyl acrylamide, which exhibited remarkable viscosity-enhancing effects in water owing to the reversible physical associations between the hydrophobic side chains of the copolymer molecules, resulting in the formation of a three-dimensional network structure. Wang et al. [59] synthesized a hydrophobically associating polymer using AM, acrylic acid (AA), 2-acrylamido-2-methylpropane sulfonic acid (AMPS), and alkyl acrylamide as raw materials. They found that the incorporation of hydrophobic monomers enhanced the intermolecular associations of the polymer, leading to improved drag reduction and viscosity enhancement capabilities. Mao et al. [60] discovered that the hydrophobic associative structure can maintain a certain degree of molecular chain extension for drag reducers in salt solutions, resulting in better viscosity enhancement. Furthermore, stronger hydrophobic associations correspond to superior viscosity enhancement effects.

Although the introduction of hydrophobic side groups onto polyacrylamide molecules effectively enhance the thickening ability of polyacrylamide-based drag reducers, it results in high viscosity during fluid handling, making pumping challenging, while exhibiting relatively poor temperature resistance.

#### 5.1.2. Grafting of Strong Polar Side Chains

The presence of salt ions in the water used for hydraulic fracturing can compress the double electron layer of the polymer molecular chain, causing the polymer chains of drag reducers to coil, resulting in a decrease in their effectiveness in reducing drag and thickening the fluid. To address this issue, strong polar side groups, such as -SO_3_H, can be introduced into the polyacrylamide molecules. When hydrolyzed, these groups generate strong electrostatic repulsion forces within and between the polymer chains, weakening the electrostatic shielding effect of the cations in saline water on polyacrylamide. At the same time, it can also synergistically work with the hydrolyzed group of acrylamide -COO^−^ to improve the rigidity of the molecular chain to reduce the degree of curling of the molecular chain. This reduces the degree of chain coiling, allowing the chains to stretch out and increase their size, thereby enhancing their ability to thicken the fluid. Additionally, this modification improves the water solubility, temperature resistance, and salt resistance of polyacrylamide [61].

**Figure 14:**
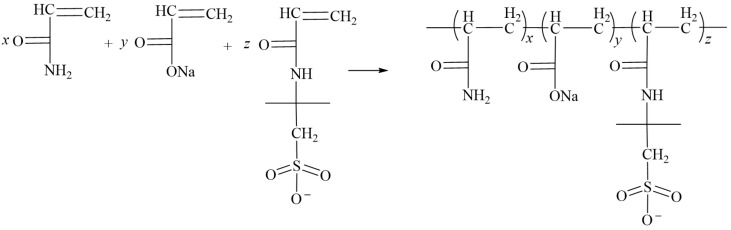
AM + AA + AMPS copolymerization equation [62].

Common monomers containing strong polar groups include acrylic acid (AA), sodium styrene sulfonate, and AMPS [56]. For instance, Li [62] synthesized a ternary emulsion drag reducer using AM, AA, and AMPS, which achieved a reduction rate of 75.6% at a concentration of 0.1 wt% and a viscosity of 0.052 Pa·s at a concentration of 0.5wt%, as shown in Figure 14. Kujawa et al. [63,64] incorporated AMPS in the synthesis of both amphiphilic acrylamide copolymers and hydrophobic associative acrylamide copolymers, both of which exhibited good thickening effects in pure water and a sodium chloride solution. Furthermore, Xu et al. [65] synthesized a ternary copolymer of AM, AMPS, and NVP, which maintained a high viscosity even at high temperatures.

The introduction of strong polar groups can effectively improve the thickening effect and temperature and salt resistance of drag reducers in saline water. However, an excessively high proportion of strong polar groups can increase steric hindrance during the polymerization process, resulting in a reduced polymerization degree and molecular weight of the product, as well as increased production costs.

#### 5.1.3. Grafting of Zwitterionic Side Chains

**Figure 15:**
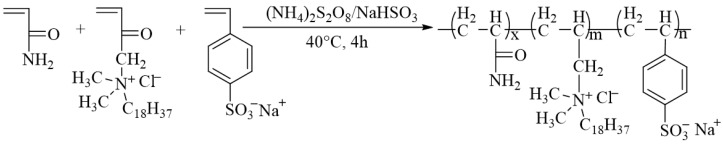
AHAPAM synthesis roadmap [69].

Introducing side groups containing both anionic and cationic moieties into polyacrylamide molecules can induce molecular chain stretching, lead to an increase in the solution’s viscosity [66]. Li et al. [67,68] synthesized a zwitterionic polymer using AM, AMPS, and long-chain alkyl acryloyloxyethyl trimethyl ammonium chloride as raw materials. The introduction of zwitterionic groups weakens the intramolecular associations of the polymer and enhances the hydrophobic association between polymer molecules through hydrogen bonding or van der Waals forces, thereby increasing the viscosity of the aqueous solution. Huang et al. [69] prepared an amphiphilic hydrophobically associating polyacrylamide (AHAPAM) by using sodium p-styrene sulfonate (SSS), N,N-dimethylarylamine chloride, and AM, as shown in Figure 15. AHAPAM exhibited excellent salt resistance, high temperature resistance, and shear resistance. At 140 °C and a shear rate of 170 s^−1^, a 0.5 wt% polymer solution maintained a viscosity above 0.092 Pa·s.

Although amphoteric polyacrylamides demonstrate good resistance to salt, their synthesis cost is relatively high.

### 5.2. Polymerization Methods

Currently, there are four primary synthesis methods for associative polyacrylamide: aqueous solution polymerization, dispersion polymerization, inverse emulsion polymerization, and micellar polymerization.

#### 5.2.1. Aqueous Solution Polymerization

**Figure 16:**
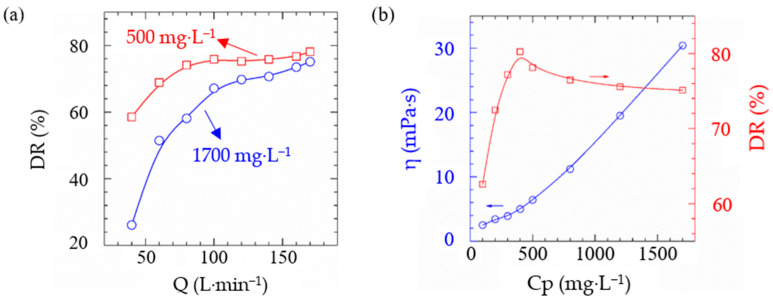
(**a**) The drag reduction rate with the displacement and (**b**) the apparent viscosity with different concentration of copolymer [72].

The polymerization reaction of monomers in water is referred to as aqueous solution polymerization, where the polymerization system consists of monomers, initiators, and water. This synthesis process is simple, has a high yield, and the resulting products are easy to transport and store [70]. Lin [71] first synthesized a hydrophobic monomer, DMDA, by reacting 2-methacryloyloxyethyl dimethylamine with 1-bromododecane. Subsequently, an AM-DMDA binary copolymer with hydrophobic associations was synthesized using aqueous solution polymerization, which exhibited excellent viscosity retention in saline water. In another study, Wei et al. [72] utilized aqueous solution polymerization to synthesize an AM-AMPS binary copolymer, which was further processed into a powdered drag reducer. At a concentration of 0.04 wt%, the drag reduction rate was 80.2% with a viscosity of 0.005 Pa·s, and at a concentration of 0.17 wt%, the drag reduction rate reached 75.1% with a viscosity of up to 0.030 Pa·s, as shown in Figure 16.

However, the products obtained from aqueous solution polymerization require purification and drying to obtain powdered drag reducers. The cost of solvent separation and recovery is high, and the product is prone to degradation during the drying process. Moreover, powdered drag reducers dissolve slowly in water and require pre-mixing, which increases the construction time. Consequently, this process increases both production and application costs.

#### 5.2.2. Dispersion Polymerization

The dispersive polymerization method is a technique derived from the precipitative polymerization method, where the polymerization system comprises monomers, organic solvents, dispersants, water-soluble initiators, and water. During the polymerization reaction, when the polymer chains reach a certain length, they precipitate from the continuous phase and form a dispersed phase. This process is facilitated by the action of dispersants, resulting in the formation of a stable dispersion system [73]. In a study conducted by Dai et al., a zwitterionic polyacrylamide was synthesized using the dispersion polymerization method with AM, AA, and methyl acryloyloxyethyl trimethyl ammonium chloride as monomers. The long polymer chains acted as bridges between particles, leading to an increase in the solution’s viscosity [74].

The resulting product from dispersion polymerization is a water-in-water emulsion drag reducer, which exhibits a rapid dissolution rate in water and create minimal environmental pollution [75]. However, the thickening effect of the product is relatively weak, and the synthesis of polyacrylamide drag reducers with long-chain hydrophobic side groups remains challenging.

#### 5.2.3. Inverse Emulsion Polymerization

**Figure 17:**
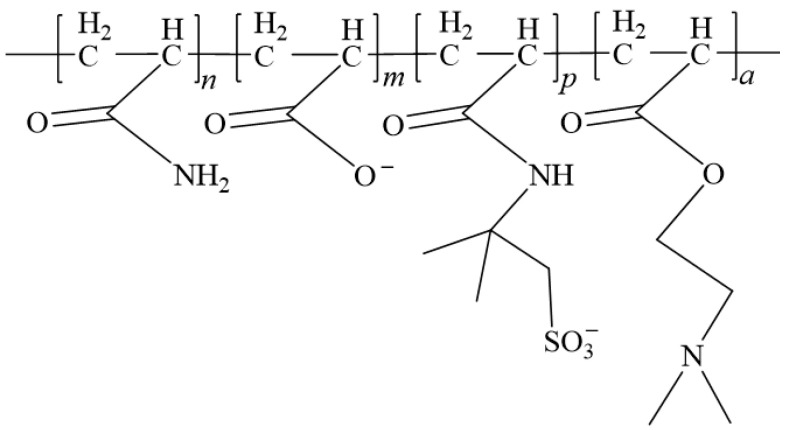
Structural formula of SFFRE-1 inverse emulsion drag reducer [79].

**Figure 18:**
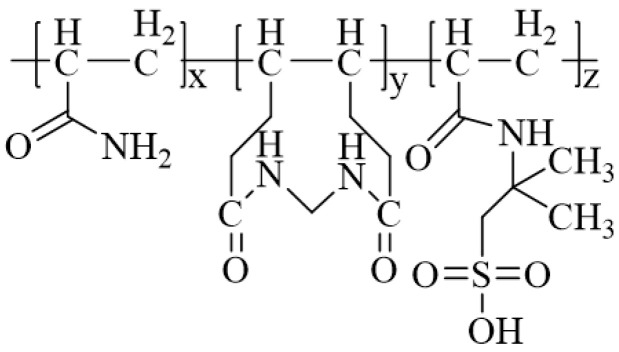
AM/AMPS/MBA copolymer [80].

In 1962, Vanderhoff introduced the concept of inverse emulsion polymerization. The polymerization system consists of monomers, organic solvents, oil-soluble initiators, oil-soluble emulsifiers, and water [76,77]. This method involves dissolving water-soluble monomers in non-polar organic solvents and conducting polymerization reactions under the influence of oil-soluble emulsifiers. Aften C et al. [78] successfully synthesized a polyacrylamide drag reducer using the inverse emulsion polymerization method, which exhibited good thickening effects even in saltwater solutions at a concentration of 0.5wt%. Wei [79] employed inverse emulsion polymerization to synthesize a viscosity-controllable drag reducer, SFFRE-1, by copolymerizing AM, AA, AMPS, and dimethylaminoethyl acrylate, as shown in Figure 17. At a concentration of 0.1wt%, the viscosity of the aqueous solution ranged from 0.002 to 0.0035 Pa·s, with a drag reduction rate of 80.0%. At a concentration of 1.0%, the viscosity reached 0.120 Pa·s, making it suitable for use in sand-carrying fluids. Zhang et al. [80] synthesized a polyacrylamide emulsion through inverse emulsion polymerization by copolymerizing AM, AMPS, and N,N-methylene bisacrylamide, as shown in Figure 18. At a concentration of 2.0 wt%, the aqueous solution maintained a viscosity of 0.220 Pa·s even after shearing at shear a rate of 170 s^−1^, indicating excellent thickening, sand-carrying, and shear-resistance abilities.

The products obtained from inverse emulsion polymerization are oil-in-water emulsion drag reducers characterized by a high molecular weight and narrow distribution [81]. However, the synthesis process requires the addition of a large amount of organic solvents, resulting in high costs and severe environmental pollution.

#### 5.2.4. Micelle Polymerization

**Figure 19:**
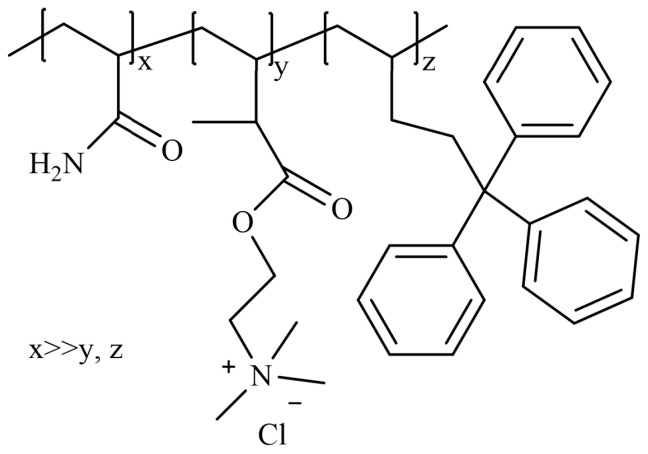
Structural formula of AM/TP/TMAEMC [82].

**Figure 20:**
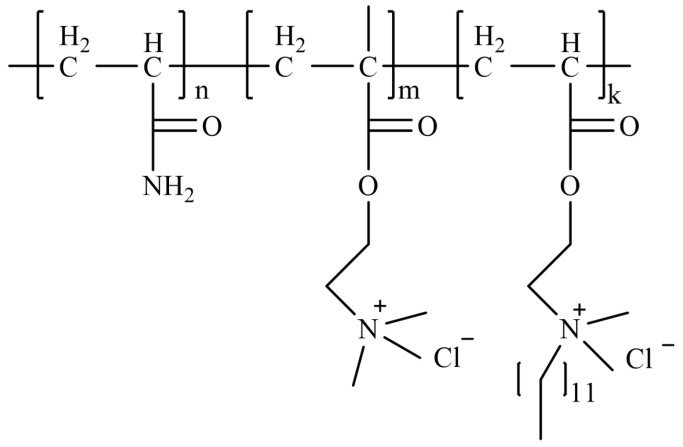
Molecular structure of water-slipping glue–liquid integrated thickener [83].

Micellar polymerization refers to the polymerization of monomers in surfactant aqueous solutions, with the polymer system comprising monomers, initiators, surfactants, and water [56,75]. This method is considered the most effective approach for preparing hydrophobically associating polyacrylamides. Zhang et al. [82] conducted micellar polymerization to synthesize hydrophobically associating ternary copolymers of AM, methyl acryloyloxyethyl trimethyl ammonium chloride (TMAEMC), and a hydrophobic monomer, 5,5,5-triphenyl-1-pentene (TP), as shown in Figure 19. The study demonstrated that the thickening effect of the copolymer critically depended on the number and length of the hydrophobic segments in the polymer chains. When the copolymer concentration exceeded 0.25 wt%, the solution viscosity reached 0.010 Pa·s. Moreover, the copolymer exhibited remarkable salt resistance, thermal stability, and shear recovery properties. Jia et al. [83] employed micellar polymerization to synthesize a viscosity-controllable drag reducer ternary copolymer of AM, methyl acryloyloxyethyl trimethyl ammonium chloride (DMC), and methyl acryloyloxyethyl dodecyl dimethyl ammonium bromide (DMDB), as shown in Figure 20. The drag reducer exhibited a dissolution time of less than 120 s, and at a concentration of 0.1 wt%, it displayed a viscosity of 0.010 Pa·s and a drag reduction rate of 65.7%.

Micellar polymerization effectively addresses the solubility challenges of hydrophobic monomers in the aqueous phase. However, the post-processing of micellar polymerization is intricate, as the surfactants employed also serve as chain terminators during free radical polymerization, hindering complete polymerization and resulting in a lower molecular weight of the polymer. Furthermore, the removal and treatment of surfactants after reaction completion pose challenges [75].

## 6. Enhancement of Fracturing Fluid Performance

### 6.1. Synergistic Application with Surfactants

The conventional approach to enhance the transport efficiency of proppants in fracturing fluids by increasing the viscosity and pumping rates often results in significant reservoir damage and escalated costs, thereby exhibiting increasingly restrictive limitations. To overcome this challenge, a number of scholars have investigated the synergistic utilization of drag reducers and surfactants to mitigate the issues associated with the cumbersome mixing of proppants and high-viscosity fracturing fluids, as well as the challenges of pumping and the formation of intricate fractures.

Jiang et al. [84] synergistically combined the surfactant ASR-1 with a drag reducer to develop a supramolecular fracturing fluid. This innovative fluid maintained a viscosity of 0.140 Pa·s even after undergoing shearing at a rate of 170 s^−1^ for 2 h at a temperature of 130 °C. Moreover, it exhibited exceptional proppant-carrying capacity and caused minimal damage to the reservoir.

Pu et al. [85] synergistically utilized β-cyclodextrin-functionalized hydrophobic associative polymers and viscoelastic surfactants as the primary constituents to develop a novel fracturing fluid, named NAF. This fluid demonstrated remarkable resistance to high temperatures and shear forces, as well as excellent proppant-carrying capabilities. Even after undergoing continuous shearing for 2 h at temperatures of 90 °C and 118 °C, the viscosity of NAF remained stable at 0.124 Pa·s and 0.058 Pa·s, respectively. Moreover, NAF produced minimal reservoir damage following the breaking of the gel structure.

Yang et al. [86] developed a fracturing fluid system known as LMPS, comprising a combination of a low molecular weight polymer (AAAM) and surfactant (SDBS). At a concentration of 0.5 wt%, the aqueous solution of LMPS exhibited a viscosity of 0.072 Pa·s and demonstrated an excellent suspension capability for 20/40 ceramic proppants. Moreover, LMPS enhanced the viscoelasticity of the fracturing fluid while maintaining a low viscosity through the interaction between hydrophobic groups on the polymer chains and the surfactant.

### 6.2. Synergistic Application with Nanomaterials

Furthermore, researchers have investigated the incorporation of nanomaterials into viscosity-controllable drag reducers, taking advantage of their functionalization and high surface activity. This combination exploits the benefits of nanomaterials, including their large specific surface area, functionalization potential, and ability to reduce interfacial tension, modify reservoir wettability, and enhance displacement capabilities. By leveraging these advantages, the stability of the drag reducers is enhanced under complex reservoir conditions characterized by high temperatures and pressures. Additionally, this integration improves the flowback rate of fracturing fluids in low-permeability reservoirs, reduces reservoir damage, and has the potential to enhance oil recovery through improved imbibition. Overall, this approach offers an effective means for enhancing production.

**Figure 21:**
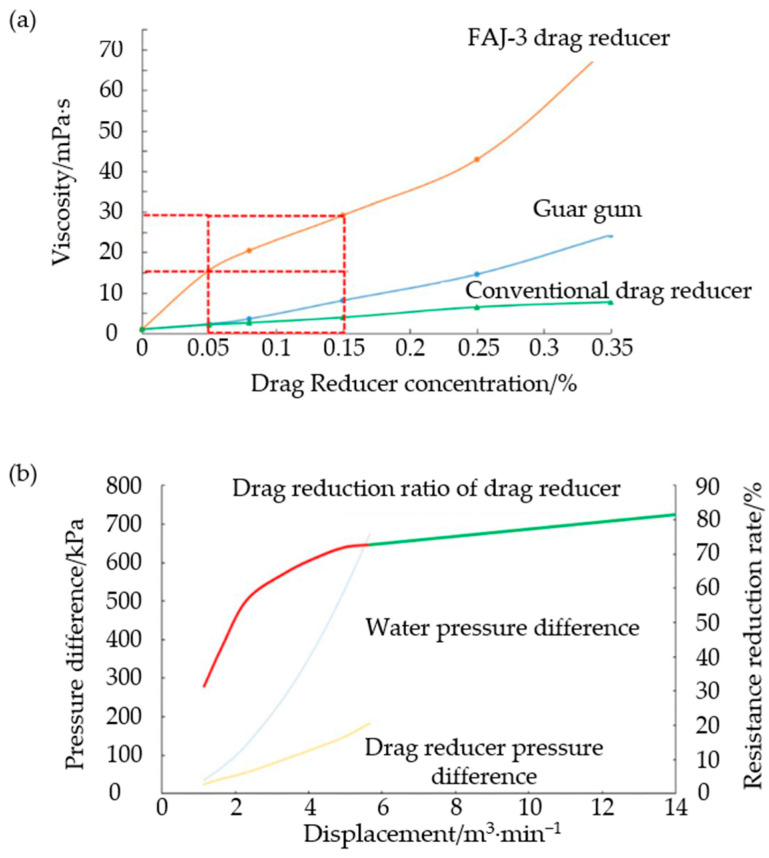
(**a**) The viscosity with different concentration of drag reducer and (**b**) the drag reduction rate with different displacement.

Xu et al. [11] developed a viscosity-controllable drag reducer through the ternary copolymerization of AM, AMPS, and dimethyl-diallylammonium chloride (DMDAAC), and incorporated nano-silica to create an innovative slickwater fracturing fluid. The interaction of supramolecular forces between the drag reducer and nano-silica significantly enhanced the slickwater’s temperature and salt resistance, as well as its shear resistance. At a concentration of 0.05 wt%, the drag reduction rate exceeded 75.0%, while a concentration of 0.2 wt% allowed for a sand ratio of up to 30% in the fracturing fluid, as shown in Figure 21.

**Figure 22:**
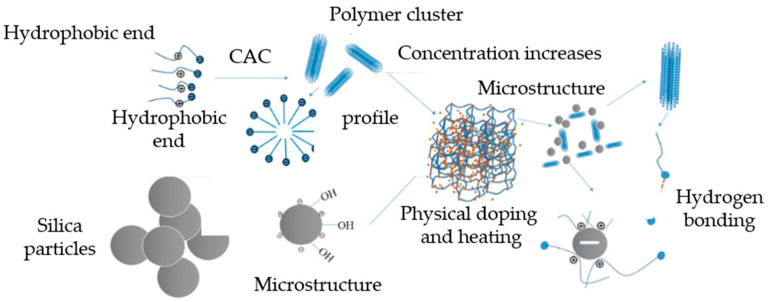
The tackifying mechanism of SiO_2_ to improve the viscosity of polymers.

Kang et al. [87] incorporated negatively charged SiO_2_ nanoparticles into hydrophobically associating polymers. The introduction of SiO_2_ nanoparticles led to a notable improvement in the apparent viscosity and viscoelasticity of the polymers. Moreover, the SiO_2_ nanoparticles acted as a supportive agent, enhancing the structural integrity between polymer molecules, as shown in Figure 22. This enhancement resulted in improved resistance to salt and high temperatures for the polymers.

Zhou et al. [88] incorporated a nanoscale emulsion additive, CNDAD#1, into fracturing fluids and observed its effective mitigation of “water blocking damage”, resulting in improved flowback rates for slickwater and working fluids following fracturing operations.

Jiang et al. [89] combined a nanoscale emulsion CND with a drag reducer (FR-900) to construct a multifunctional fracturing fluid that possesses the capabilities of “fracturing, energy enhancement, and oil displacement”. This fluid was successfully applied in the Changqing oilfield in China. The maximum proppant-carrying capacity reached 400 kg/m^3^, resulting in a production rate of up to 26.5 t/d after completion, effectively enhancing the oil recovery capacity.

## 7. Conclusions

This article provides an overview of the evolution of polymer drag reducers for hydraulic fracturing. It discusses the synthesis routes and methods of viscosity-controllable polyacrylamide-based drag reducers, and summarizes their thickening mechanisms. The article also presents the application status of these drag reducers and highlights their advantages over conventional slickwater fracturing fluids. The key findings are as follows:

(1) Compared with linear gel fracturing fluids and crosslinked gel fracturing fluids, viscous slick water fracturing fluids have the functions of reducing resistance and making fractures, increasing viscosity, and carrying sand, which simplifies the field pumping process, reduces the amount of chemical agent needed, and reduces the cost of fracturing operations.

(2) Viscosity-controllable drag reducers can be synthesized by incorporating hydrophobic side groups, strong polar groups, and zwitterionic groups into polyacrylamide molecules, thereby enhancing their thickening effects.

(3) The synthesis of viscosity-controllable polyacrylamide-based drag reducers involves various methods, including solution polymerization, dispersion polymerization, inverse emulsion polymerization, and micellar polymerization. Among these methods, inverse emulsion polymerization and micellar polymerization are particularly suitable for synthesizing drag reducers with long-chain hydrophobic side groups.

(4) Viscosity-controllable slick water fracturing fluids can be used together with surfactants and nanomaterials to form integrated methods for fracturing and enhanced oil recovery.

(5) The usage of large water volumes in hydraulic fracturing operations for shale gas reservoirs poses a risk of gas flow channel blockages, ultimately resulting in reduced well productivity and potential groundwater contamination. Thus, there is a demand for drag reducers that result in less damage and are environmentally friendly.

## 8. Discussion

Shale gas resources have great potential, but deep/ultra-deep reservoirs have higher requirements for fracturing technology and equipment. The viscosity of viscosity-controllable fracturing fluids can be adjusted in real time, so as to realize ‘low viscosity for drag reduction and high viscosity for sand-carrying’, which can effectively reduce the cost of fracturing. In addition, through the research on fracture detection technology, the design of fracturing construction can be optimized and the pertinence of fracturing schemes can be improved, which provides a basis for the development of fracturing and improves the efficiency of fracturing construction.

## Data Availability

Data are contained within the article.
